# Supplementary material for: Thermal-intensified interfacial polymerization enables ultra-selective reverse osmosis membrane for toxic micropollutant removal
Source: Nat Commun. 2025 Oct 9;16:9004. doi: 10.1038/s41467-025-64056-z (PMC12511291; doi:10.1038/s41467-025-64056-z)
Supplement: Supplementary file 1 — Supplementary Information [file 41467_2025_64056_MOESM1_ESM.pdf]

Supplementary information

Thermal-intensified interfacial polymerization enables ultra-selective reverse osmosis membrane for toxic micropollutant removal

Shenghua Zhou,[1] Lu Elfa Peng,[1] Wenyu Liu,[1] Hao Guo,[2, 3]* Chuyang Y. Tang[1]*

[1] Department of Civil Engineering, The University of Hong Kong, Pokfulam, Hong Kong SAR 999077, China.

[2] Institute of Environment and Ecology, Shenzhen International Graduate School, Tsinghua University, Shenzhen 518055, China.

[3] Guangdong Provincial Key Laboratory of Carbon Fixation and Sinks, Department of Education of Guangdong Province, Shenzhen International Graduate School, Tsinghua University, Shenzhen 518055, China.

* Corresponding authors:

Dr. Hao Guo (Email address: guohao@sz.tsinghua.edu.cn)

Prof. Chuyang Y. Tang (Email address: tangc@hku.hk)

Number of pages (including the cover page): 41

Number of figures: 27

Number of tables: 7

Table of contents

Supplementary Note 1. Effects of high-temperature Isopar G on the substrate…………Page S3
Supplementary Note 2. Interfacial polymerization (IP) reaction.…………..….………..Page S4
Supplementary Note 3. Ultraviolet-visible (UV) absorbance of MPD……………….…Page S5
Supplementary Note 4. Detection of MPD diffusion……………………..……..…... Page S6
Supplementary Note 5. Fourier transform infrared (FTIR) spectra of thermal-intensified IP (TIP) membrane…………………………………………………..…………....…Page S7
Supplementary Note 6. X-ray photoelectron spectroscopy (XPS) characterization of TIP membranes……………………………………………………………………..….Page S8
Supplementary Note 7. Normalized carboxyl group density of TIP membranes……....Page S11
Supplementary Note 8. Water contact angle (WCA) of TIP membranes……...…...….Page S11
Supplementary Note 9. Effects of post-heating on the TIP membranes……..………....Page S12
Supplementary Note 10. Pore size distribution of TIP membranes….…...………..……Page S13
Supplementary Note 11. Evaluation of gas/vapor generation during IP reaction……...Page S15
Supplementary Note 12. Measurement of back pore size for TIP membranes..…....…Page S16
Supplementary Note 13. Calculation of nanovoid fraction for TIP membranes …..…..Page S18
Supplementary Note 14. Evaluation of structural stability of nanovoids…..……..……Page S19
Supplementary Note 15. Apparent thickness and intrinsic thickness of a polyamide membrane……………………………………………………………………..….…Page S20
Supplementary Note 16. Average roughness of TIP membranes…………………..….Page S24
Supplementary Note 17. UV-based humic acid (HA) determination…..………..…..…Page S25
Supplementary Note 18. Flux distribution of TIP membranes……………..………..…Page S26
Supplementary Note 19. Molecular dynamics (MD) simulation of monomer diffusion
…………………………………………………………………………………Page S27
Supplementary Note 20. Density functional theory (DFT) calculation of IP reaction…Page S30
Supplementary Note 21. Chemical information about micropollutants………………..Page S31
Supplementary Note 22. Rejection of micropollutants by polyamide membranes…….Page S32
Supplementary references……………………………………………………….…..Page S38

Supplementary Note 1. Effects of high-temperature Isopar G on the substrate

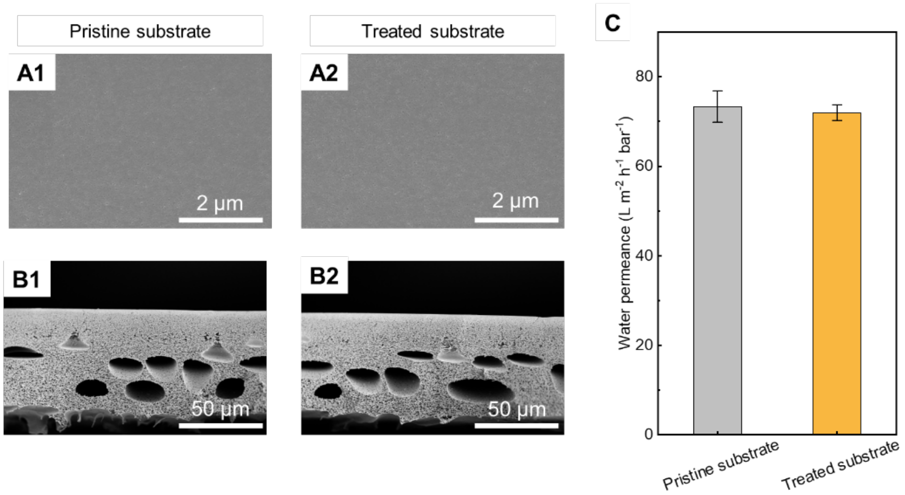

Supplementary Fig. 1| (**A**) SEM surface morphology, (**B**) SEM cross-sectional structures, and (**C**) water permeance of the pristine PSf substrate and the substrate immersed in 100 °C Isopar G for 1 min. Filtration test conditions: applied pressure of 1 bar; crossflow velocity of 22.4 cm s^{-1}; pure water as the feed solution; pre-compaction time of 2 h. The error bars represent the standard deviation of the results obtained from at least three independent measurements of different membranes.

SEM characterization does not show obvious changes for the PSf substrate after being immersed in the 100 °C Isopar G (Supplementary Fig. 1A and Fig. 1B). Moreover, the water permeance of the treated substrate (72.0 ± 1.7 L m^{-2} h^{-1} bar^{-1}) was comparable to that of the pristine substrate (73.3 ± 3.5 L m^{-2} h^{-1} bar^{-1}, Supplementary Fig. 1C). These results suggested that high-temperature Isopar G does not significantly alter the structure and properties of the substrate.

Supplementary Note 2. Interfacial polymerization (IP) reaction

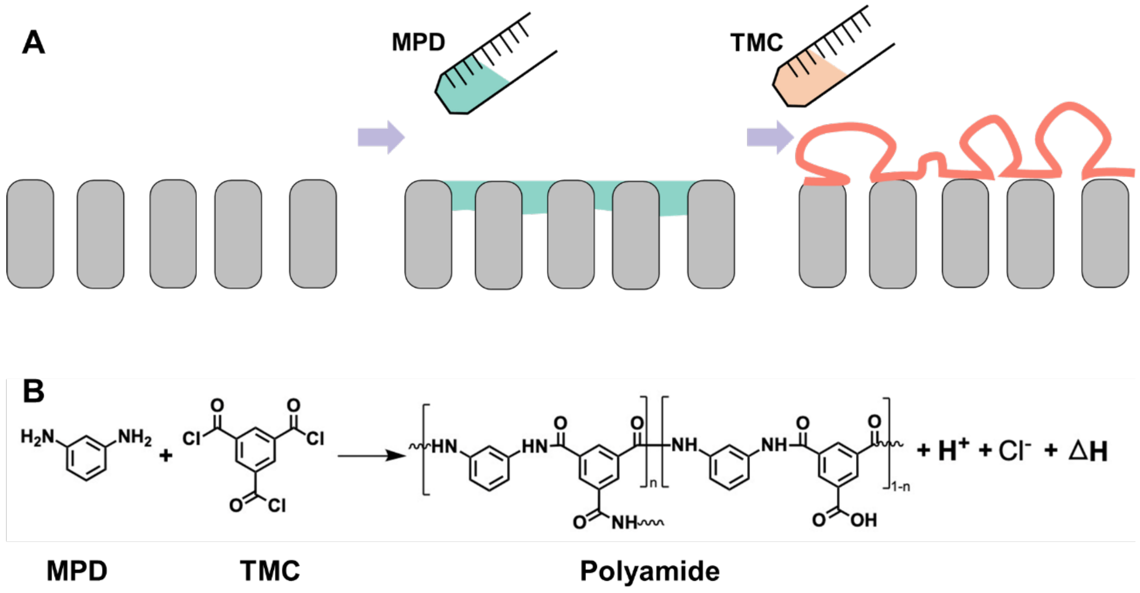

Supplementary Fig. 2| (**A**) Schematic illustration of reverse osmosis (RO) membrane fabrication by an IP reaction. (**B**) IP reaction between *m*-phenylenediamine (MPD) and trimesoyl chloride (TMC).

As shown in Supplementary Fig. 2A, the IP reaction was performed on a porous polysulfone substrate (PSf, molecular weight cut-off of 67 kDa). Briefly, a 30 mL aqueous solution containing 2 wt.% MPD was poured onto the PSf substrate for 2 min. Subsequently, the MPD was removed by a rubber roller, followed by the addition of a 30 mL Isopar G solution containing 0.1 wt.% TMC at certain temperatures (i.e., 0, 25, 50, or 100 °C, respectively) to initiate the IP reaction for 1 min. This reaction generates a fully aromatic polyamide together with byproducts of acid and heat (Supplementary Fig. 2B).

Supplementary Note 3. Ultraviolet-visible (UV) absorbance of MPD

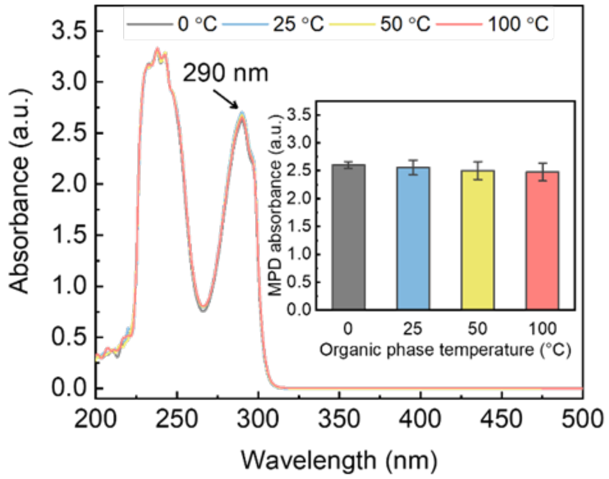

Supplementary Fig. 3| The UV absorbance spectra of MPD in Isopar G at different temperatures with wavelengths ranging from 200 to 500 nm. The inset is the UV absorbance at 290 nm at different temperatures. The error bars represent the standard deviation of the results obtained from at least three independent measurements.

We further analyzed the UV absorbance spectra of MPD in Isopar G at different temperatures (i.e., 0 °C, 25 °C, 50 °C, and 100 °C). Briefly, 2 g MPD monomers were first added into 50 mL Isopar G at room temperature (i.e., 25 °C), followed by an ultrasound mixing for 30 min. A 3 mL of the upper solution was then taken, and its temperature was adjusted to the target temperature (0 °C, 25 °C, 50 °C, or 100 °C) through cooling or heating. Subsequently, UV absorbance spectra of MPD in Isopar G at each temperature were measured (Supplementary Fig. 3), using that of pure Isopar G as the blank. An identical absorbance peak at ~290 nm with similar intensity was observed for MPD in Isopar G at different temperatures. These results suggested that temperature has no obvious effect on the UV absorbance of MPD monomers in Isopar G.

Supplementary Note 4. Detection of MPD diffusion

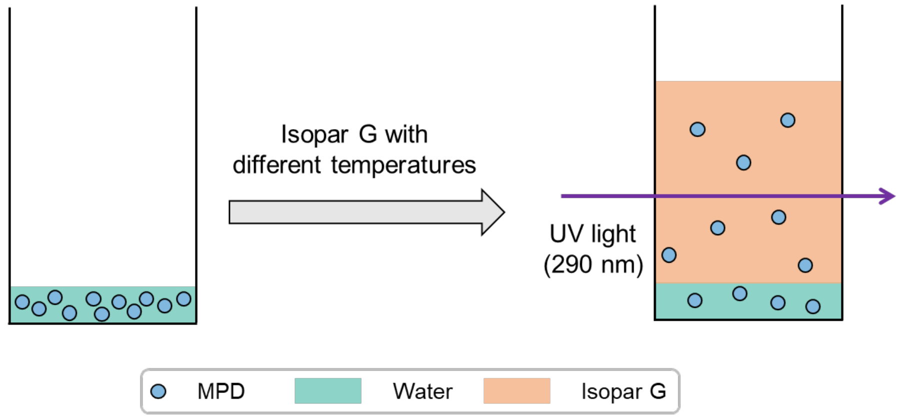

Supplementary Fig. 4| Schematic diagram of detecting MPD diffusion from water to Isopar G.

The diffusion of MPD monomers was measured using a UV spectrophotometer at a wavelength of 290 nm (Supplementary Fig. 3). Briefly, a 0.3 mL 2 wt. % MPD aqueous solution was first injected into a UV quartz cuvette (Supplementary Fig. 4). Subsequently, a 2.7 mL Isopar G solution at various temperatures (0 °C, 25 °C, 50 °C, or 100 °C, respectively) was carefully added on top of the MPD solution along the inner wall of the quartz cuvette using a pipette. After a duration of 1 min (corresponding to the IP reaction time), the MPD absorbance signal was measured for the organic phase (Isopar G).

Supplementary Note 5. Fourier transform infrared (FTIR) spectra of thermal-intensified IP (TIP) membranes

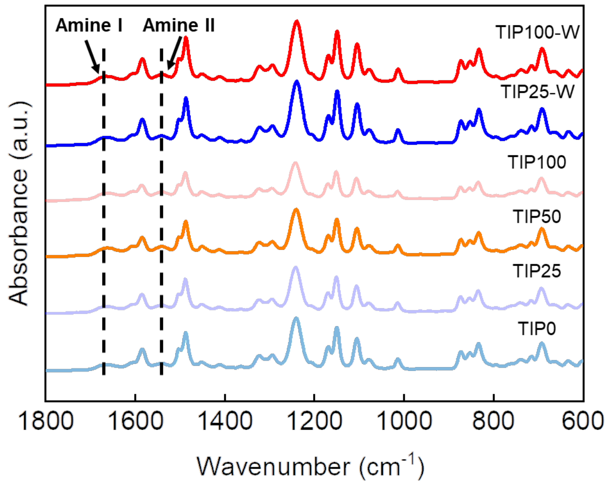

Supplementary Fig. 5| FTIR spectra of various TIP membranes.

The characteristic peak of FTIR spectra at 1663 cm^{-1} and 1541 cm^{-1} (Supplementary Fig. 5) can be ascribed to amine I and amine II band,[1] indicating the successful synthesis of fully aromatic polyamide.

Supplementary Note 6. X-ray photoelectron spectroscopy (XPS) characterization of TIP membranes

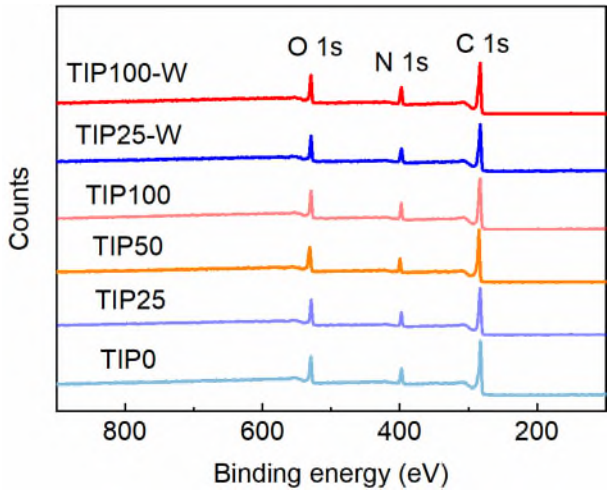

Supplementary Fig. 6| X-ray photoelectron spectroscopy (XPS) spectra of various TIP membranes.

Supplementary Table 1| C, O, N atomic contents and O/N ratio of TIP membranes. The error bars represent the standard deviation of the results obtained from at least three independent measurements of different membranes.

**Membrane**	**C (%)**	**O (%)**	**N (%)**	**O/N ratio**
TIP0	73.8 ± 0.1	16.7 ± 0.1	9.5 ± 0.2	1.76 ± 0.03
TIP25	73.8 ± 0.2	15.5 ± 0.1	10.7 ± 0.2	1.45 ± 0.03
TIP50	74.1 ± 0.3	14.5 ± 0.3	11.4 ± 0.1	1.27 ± 0.02
TIP100	72.5 ± 1.3	14.7 ± 0.7	12.8 ± 0.7	1.16 ± 0.04
TIP25-W	74.9 ± 0.1	14.0 ± 0.1	11.1 ± 0.1	1.26 ± 0.02
TIP100-W	74.5 ± 0.1	13.2 ± 0.2	12.3 ± 0.1	1.08 ± 0.02

The chemical composition and O/N ratio of polyamide layer was determined based on XPS characterization (Supplementary Fig. 6 and Table 1). The O/N ratio of TIP membranes dropped greatly at higher reaction temperature, indicating improved crosslinking degree resulting from intensified IP reactions. In addition, post-heating further decreased O/N ratio compared with their counterpart without heat treatment, which could be attributed to secondary crosslinking induced by heat.

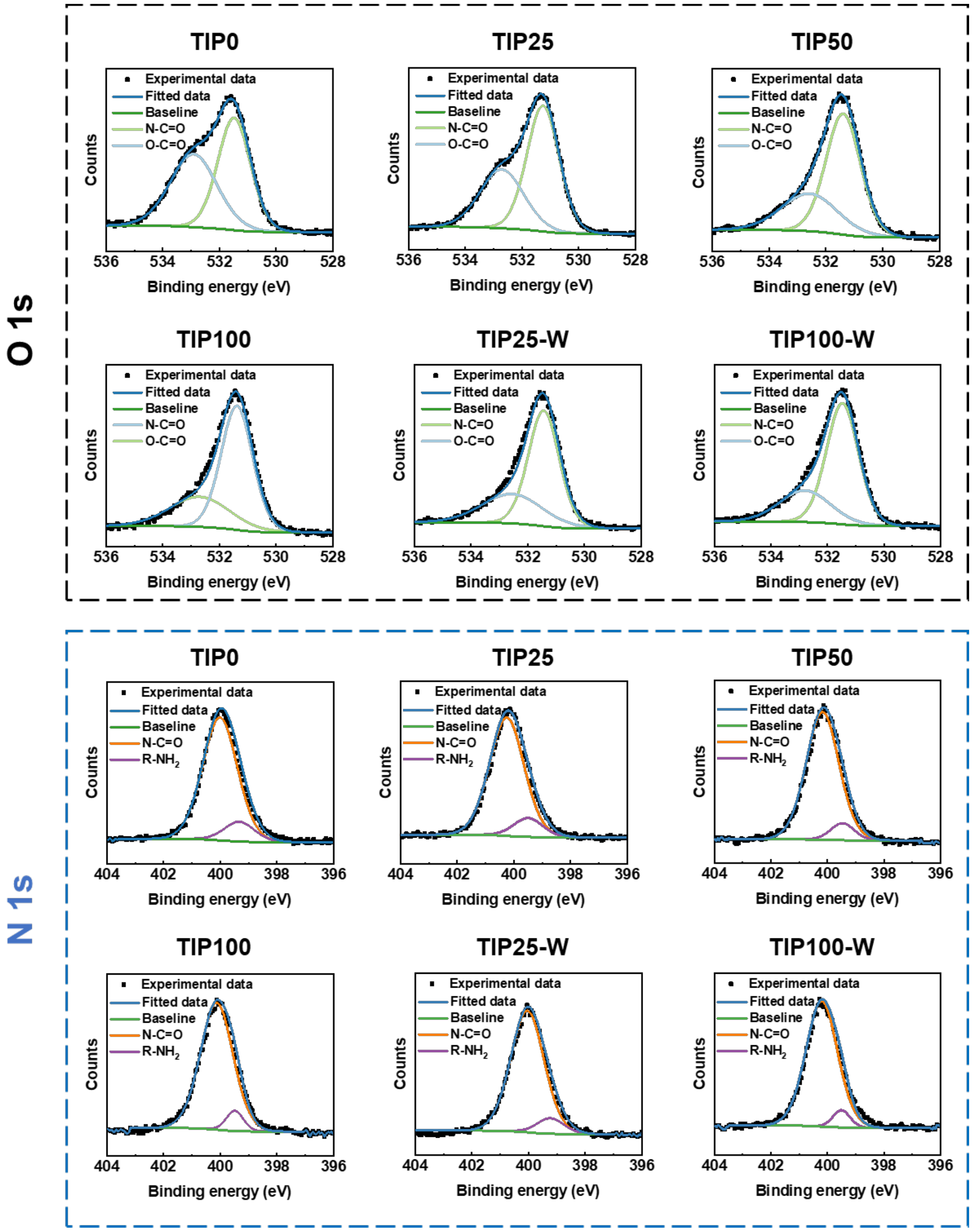

Supplementary Fig. 7| High-resolution XPS spectra of O 1s and N 1s for various TIP membranes. The amide group (N-C=O) and carboxylic group (O-C=O) in the O 1s spectrum were assigned to the peak at ~531.4 eV and ~532.7 eV, respectively. Meanwhile, unreacted amine groups (R-NH_2) and the amide group (N-C=O) in the N 1s spectrum were assigned to ~399.4 eV and ~400.2 eV, respectively.

We further analyzed the O 1s and N 1s peaks obtained from the high-resolution XPS spectra of various TIP membranes (Supplementary Fig. 7 and Table 2). Notably, the TIP membrane

formed at higher reaction temperature showed enhanced amide group content, while the value of the carboxylic group and unreacted amine reduced. This result indicates the formation of a more crosslinked polyamide. Moreover, post-heating further increased amide group content and decreased content of carboxylic and unreacted amide groups as a result of the heat-induced secondary crosslinking of polyamide.

Supplementary Table 2| XPS results of various TIP membranes. Binding energies and plausible species were determined from the O 1s and N 1s XPS spectra.

**Membrane**	**O 1s**			**N 1s**		
	Energy (eV)	Species	(%)	Energy (eV)	Species	(%)
TIP0	531.4	N-C=O	51.1	399.4	R-NH_2	18.8
	532.7	O-C=O	48.9	400.2	N-C=O	81.2
TIP25	531.4	N-C=O	53.0	399.4	R-NH_2	16.4
	532.7	O-C=O	47.0	400.2	N-C=O	83.6
TIP50	531.4	N-C=O	60.2	399.4	R-NH_2	15.5
	532.7	O-C=O	39.8	400.2	N-C=O	84.5
TIP100	531.4	N-C=O	67.1	399.4	R-NH_2	13.7
	532.7	O-C=O	32.9	400.2	N-C=O	86.3
TIP25-W	531.4	N-C=O	60.4	399.4	R-NH_2	14.7
	532.7	O-C=O	39.6	400.2	N-C=O	85.3
TIP100-W	531.4	N-C=O	72.5	399.4	R-NH_2	10.6
	532.7	O-C=O	27.5	400.2	N-C=O	89.4

Supplementary Note 7. Normalized carboxyl group density of TIP membranes

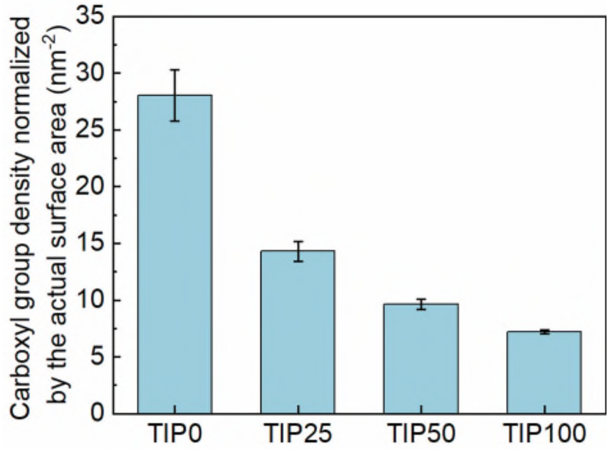

Supplementary Fig. 8| Carboxyl group density of TIP membranes normalized by the actual membrane surface area. The error bars represent the standard deviation of the results obtained from at least three independent measurements of different membranes.

Supplementary Note 8. Water contact angle (WCA) of TIP membranes

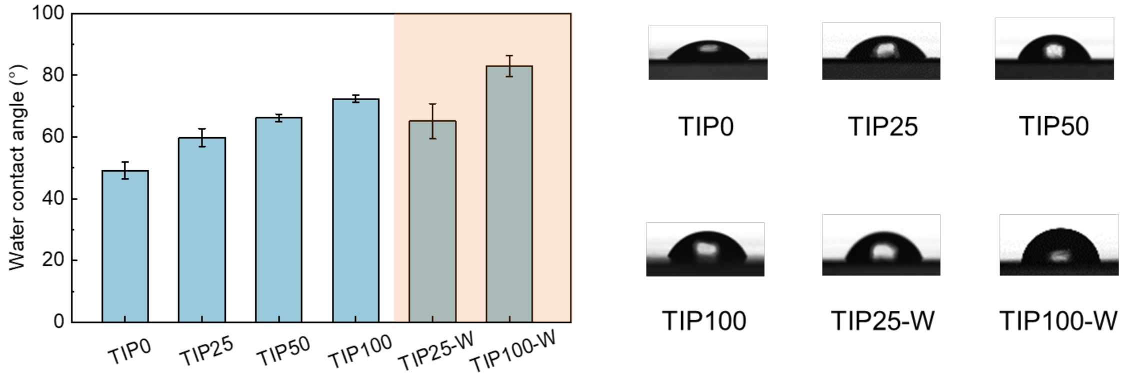

Supplementary Fig. 9| WCA of various TIP membranes. The error bars represent the standard deviation of the results obtained from at least three independent measurements of different membranes.

As shown in Supplementary Fig. 9, the WCA increased from 49.1° for TIP0 to 72.4° for TIP100. This change can be attributed to less hydrophilic ionized carboxyl groups at higher crosslinking degree (**Fig. 2**). Meanwhile, a mild increase in WCA was observed for TIP25-W compared to TIP25, while the hydrophobicity of TIP100-W was much higher than that of TIP100.

Supplementary Note 9. Effects of post-heating on the TIP membranes

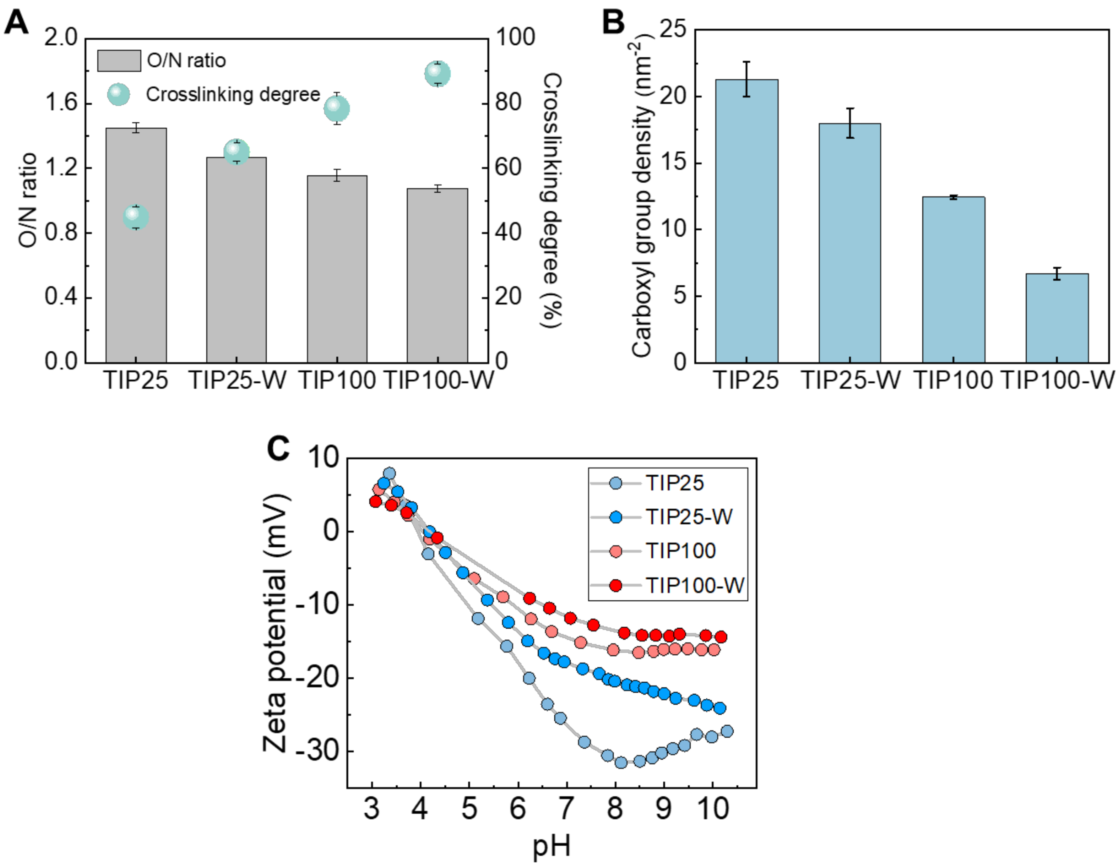

Supplementary Fig. 10| Physicochemical properties of TIP membranes with/without post-heating. (**A**) Crosslinking degree, (**B**) ionized carboxyl group density and (**C**) zeta potential. The error bars represent the standard deviation of the results obtained from at least three independent measurements of different membranes.

The polyamide crosslinking degree of TIP membranes significantly increased (Supplementary Fig. 10A) after post-heating in a 50 °C water bath for 10 min due to the heat-induced secondary crosslinking. Correspondingly, the density of ionized carboxyl groups within the polyamide reduced (Supplementary Fig. 10B), leading to less negatively charged membrane surfaces (Supplementary Fig. 10C).

Supplementary Note 10. Pore size distribution of TIP membranes

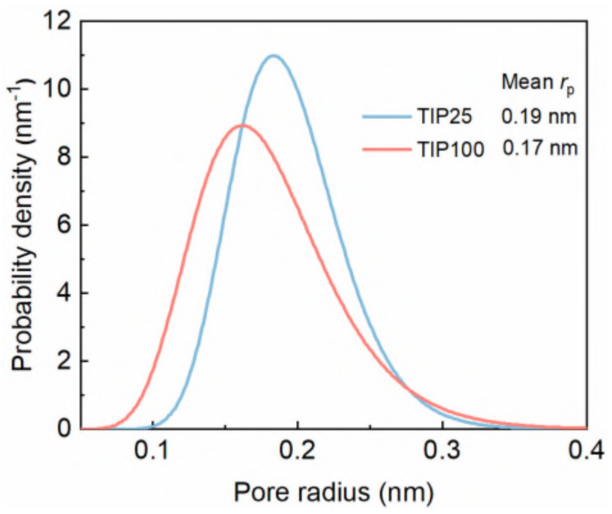

Supplementary Fig. 11| Pore size distribution of TIP25 and TIP100 membranes.

We evaluated the pore size distribution of TIP25 and TIP100 membranes based on their rejection of neutral solutes (**Fig. 2F**) with different molecular weight (Mw, g mol^{-1}), following the reported method.[2-4] Briefly, rejection of ethanol (46.1 g mol^{-1}), ethylene glycol (62.1 g mol^{-1}), glycerol (92.1 g mol^{-1}), and glucose (180.2 g mol^{-1}) was evaluated (**Fig. 2F**). The rejection for each molecule was plotted against the corresponding molecular radius (r_s, nm), with r_s calculated by the following equation:[5]

$$log r_s = -1.3363 + 0.395 \times log_{10} Mw \qquad \text{(S1)}$$

By assuming a log-normal pore size distribution, the rejection-molecular radius plot was fitted using the “log-normal CDF” function in the *Origin* software. The pore size distribution of the polyamide membrane was calculated by the probability density function:[2, 3]

$$\frac{dF(r_p)}{dr_p} = \frac{1}{r_p \rho \sqrt{2\pi}} \exp\left[-\frac{(\ln r_p - \ln \mu_p)^2}{2\sigma^2}\right] \qquad \text{(S2)}$$

where r_p is the membrane pore radius (nm) and μ_p refers to the geometric mean radius (nm) of the solute at 50% rejection, while σ denotes the geometric standard deviation calculated as ($ln\mu_p - ln\mu_d$), with μ_d representing the geometric mean radius (nm) of the solute corresponding to 84.13% rejection.

The TIP100 membrane showed a broader pore size distribution (Supplementary Fig. 11), which could be attributed to the faster reaction kinetics at elevated temperatures.[6] Nevertheless, this membrane had a smaller average pore radius of 0.17 nm, with majority of pores smaller than those of TIP25. The smaller average pore size of TIP100, beneficial for enhancing membrane rejection, can be explained by intensified IP reaction and thus higher crosslinking degree (**Fig. 2B**) at higher temperature.

Supplementary Note 11. Evaluation of gas/vapor generation during IP reaction

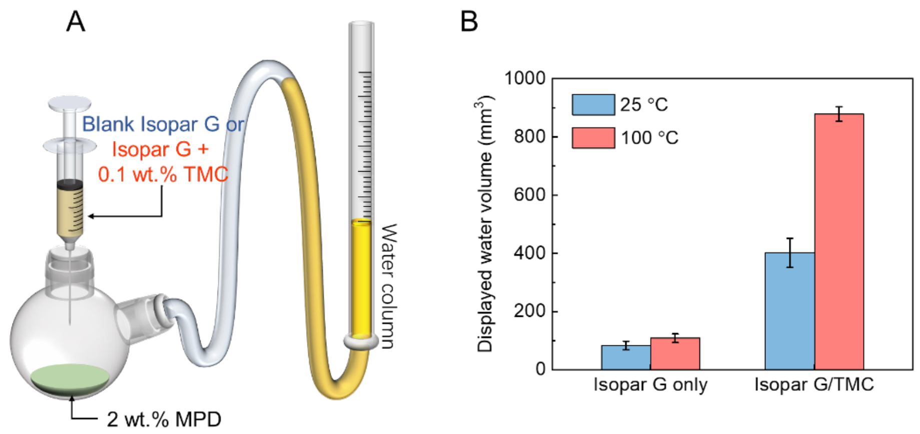

Supplementary Fig. 12| (**A**) Schematic diagram of the custom-made setup for evaluating gas/vapor generation. The airtight flask is pre-filled with 5 mL MPD (2 wt.%) and connected to a water column for the measurement of gas/vapor production. This figure was modified from Reference 7 with copyright permission. (**B**) Displaced water volume resulting from the addition of Isopar G (1 mL) at different temperatures (25 and 100 °C), without or with TMC (0.1 wt.%). The results were recorded after the system returned to the room temperature (~ 25 °C) to eliminate the effect of thermal expansion of internal gases caused by high temperature. The error bars represent the standard deviation of the results obtained from at least three independent measurements.

To further verify the interfacial degassing, we measured the volume of released gas through the displacement of water column in a custom-designed device (Supplementary Fig. 12A).[7] Compared to the addition of Isopar G only, the addition of Isopar G with TMC resulted in much greater displacement (Supplementary Fig. 12B), which demonstrates the interfacial degassing during the IP reaction. Moreover, the Isopar G/TMC solution with a higher temperature led to an increased displaced volume, which can be explained by the promoted degassing/vaporization due to the intensified IP reaction[8, 9] and the reduced gas solubility at the higher temperature. It is worthwhile noting that the 100 °C Isopar G may also accelerate the water evaporation, thereby leading to more released vapor (Supplementary Fig. 12B). The greatly enhanced gas/vapor generation, upon the encapsulation by the nascent polyamide, enhances nanovoid formation.[7, 10]

Supplementary Note 12. Measurement of back pore size for TIP membranes

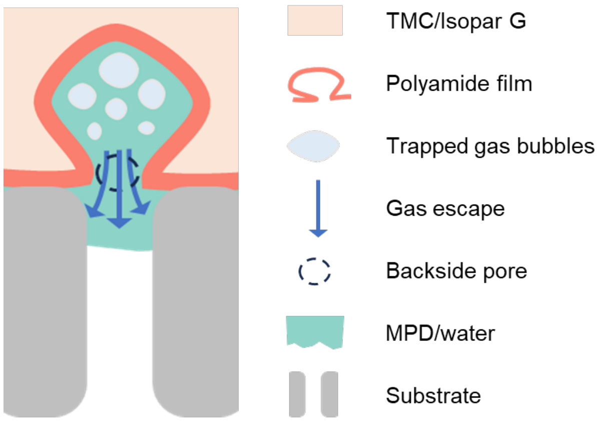

Supplementary Fig. 13| Schematic diagram of back-side pore formation during the IP reaction.

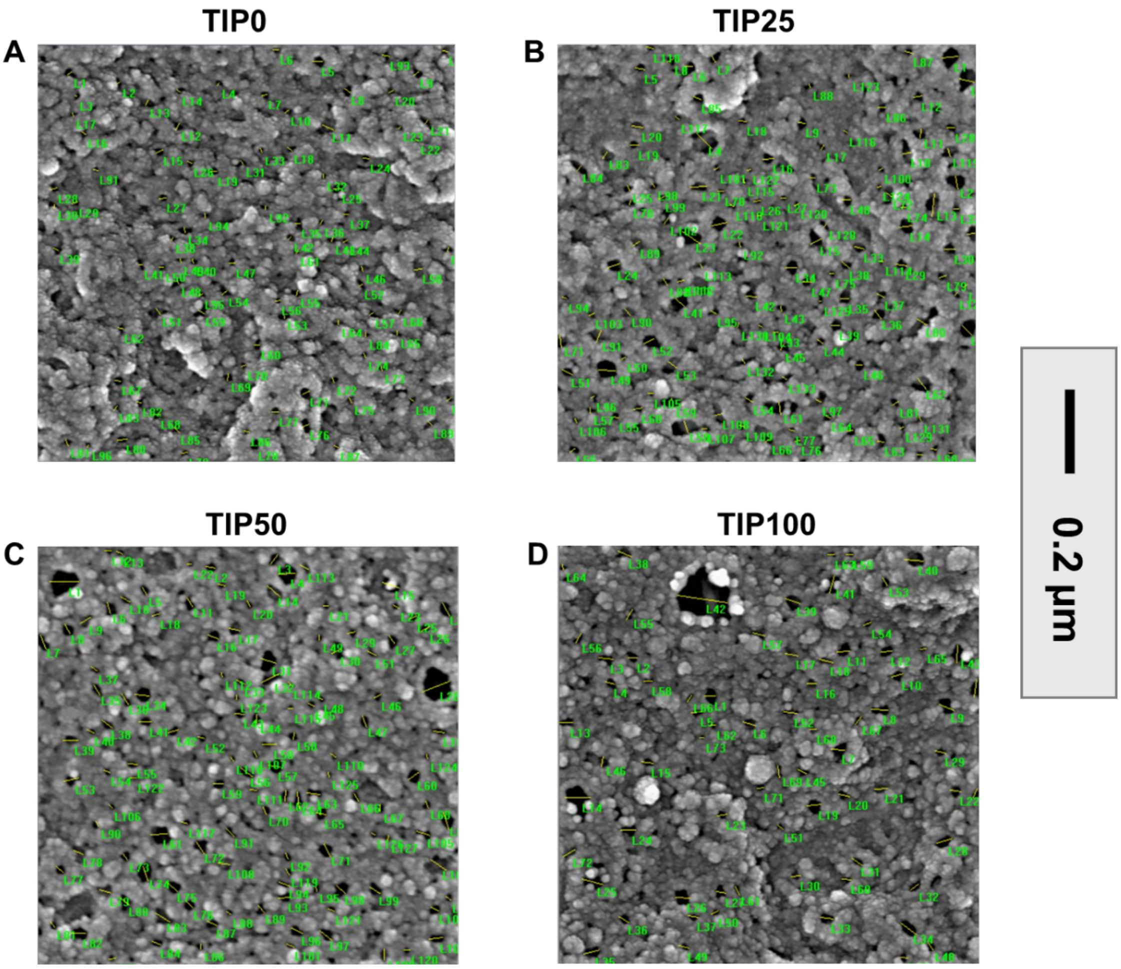

Supplementary Fig. 14| Back pore size measurement based on the SEM images of (**A**) TIP0, (**B**) TIP25, (**C**) TIP50, and (**D**) TIP100 membranes using the software of *Image-Pro Plus*. At least 70 pores were counted to determine the mean back pore size.

To analyze the back pore size of TIP membranes, the polysulfone substrate was completely dissolved by dimethylformamide. The isolated polyamide layer was then transferred onto a silicon wafer with the top surface facing the silicon wafer for SEM characterization. The back pore size was further analyzed using the software of *Image-Pro Plus* (Supplementary Fig. 14).

Supplementary Note 13. Calculation of nanovoid fraction for TIP membranes

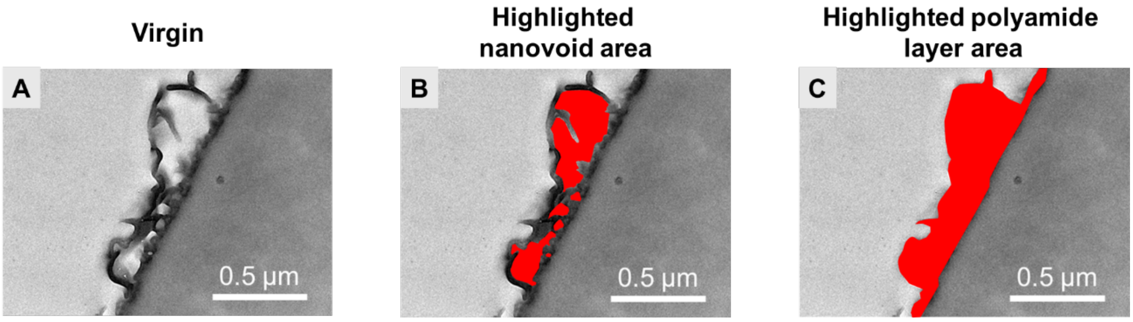

Supplementary Fig. 15| Calculation of nanovoid fraction of TIP100 membrane based on its TEM cross-section image. (**A**) The original TEM cross-section image. (**B**) The highlighted interior nanovoids (red color) within the polyamide layer. (**C**) The entire area includes the polyamide layer and the interior nanovoids. These images were analyzed by the software of *Image-Pro Plus*.

We used the TEM cross-section image of the TIP membrane (e.g., TIP100, Supplementary Fig. 15A) to show the determination of its nanovoid fraction, which is defined as the ratio of interior nanovoid area (Supplementary Fig. 15B) over the total area including polyamide layer and interior nanovoids (Supplementary Fig. 15C). The area is determined by the software of *Image-Pro Plus*.

Supplementary Note 14. Evaluation of structural stability of nanovoids

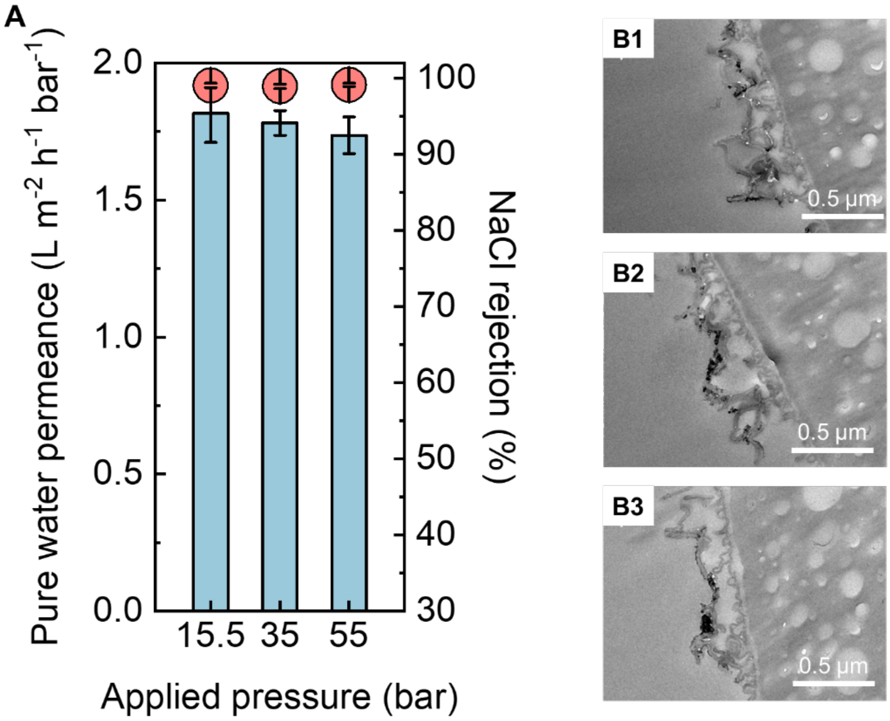

Supplementary Fig. 16. (**A**) Pure water permeance and NaCl rejection of TIP100 membranes measured at 15.5, 35, and 55 bar, respectively. The membrane was pre-compacted with pure water at the specified pressure for 2 h, followed by the pure water flux measurement. Subsequently, 2 g L^{-1} NaCl solution was used as the feed solution, and NaCl rejection was determined after another 2-h filtration at the same pressure. After the filtration tests, membrane samples were rinsed with pure water for further TEM characterization. (**B**) TEM cross-sectional morphologies of the TIP100 membrane after filtration test at 15.5 bar (**B1**), 35 bar (**B2**), and 55 bar (**B3**). The error bars represent the standard deviation of the results obtained from at least three independent measurements of different membranes.

To evaluate the structural stability of nanovoids within the polyamide membranes, we performed additional filtration tests for TIP100 membranes at 15.5, 35, and 55 bar, respectively (Supplementary Fig. 16A). The NaCl rejection of the membrane was well maintained. The slight decrease in water permeance can be attributed to membrane compaction at higher applied pressure.[11, 12] Meanwhile, the nanovoid-containing structure was still observed even after the high-pressure filtration under 55 bar (Supplementary Fig. 16B). This result is also consistent with literature.[11]

Supplementary Note 15. Apparent thickness and intrinsic thickness of a polyamide membrane

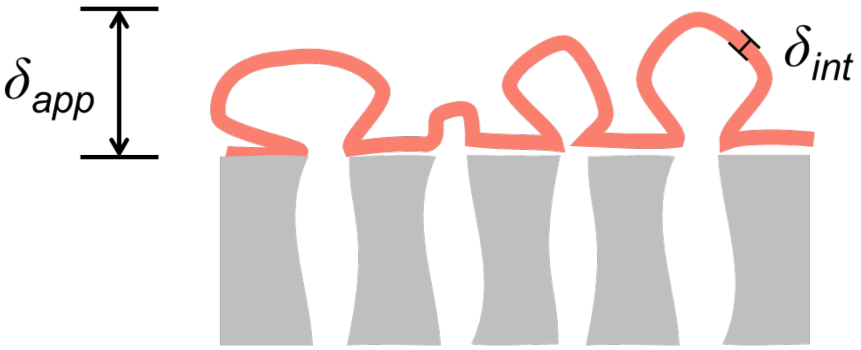

Supplementary Fig. 17| Schematic illustration of apparent thickness (δ_{app}) and intrinsic thickness (δ_{int}) of a polyamide film.

The thickness of a polyamide film can be classified as apparent thickness and intrinsic thickness (Supplementary Fig. 17).[10] The intrinsic thickness is defined as the average thickness of the nodule or leaf walls, while the apparent thickness is defined as the average vertical distance that encompasses the entire polyamide and nanovoid structures.

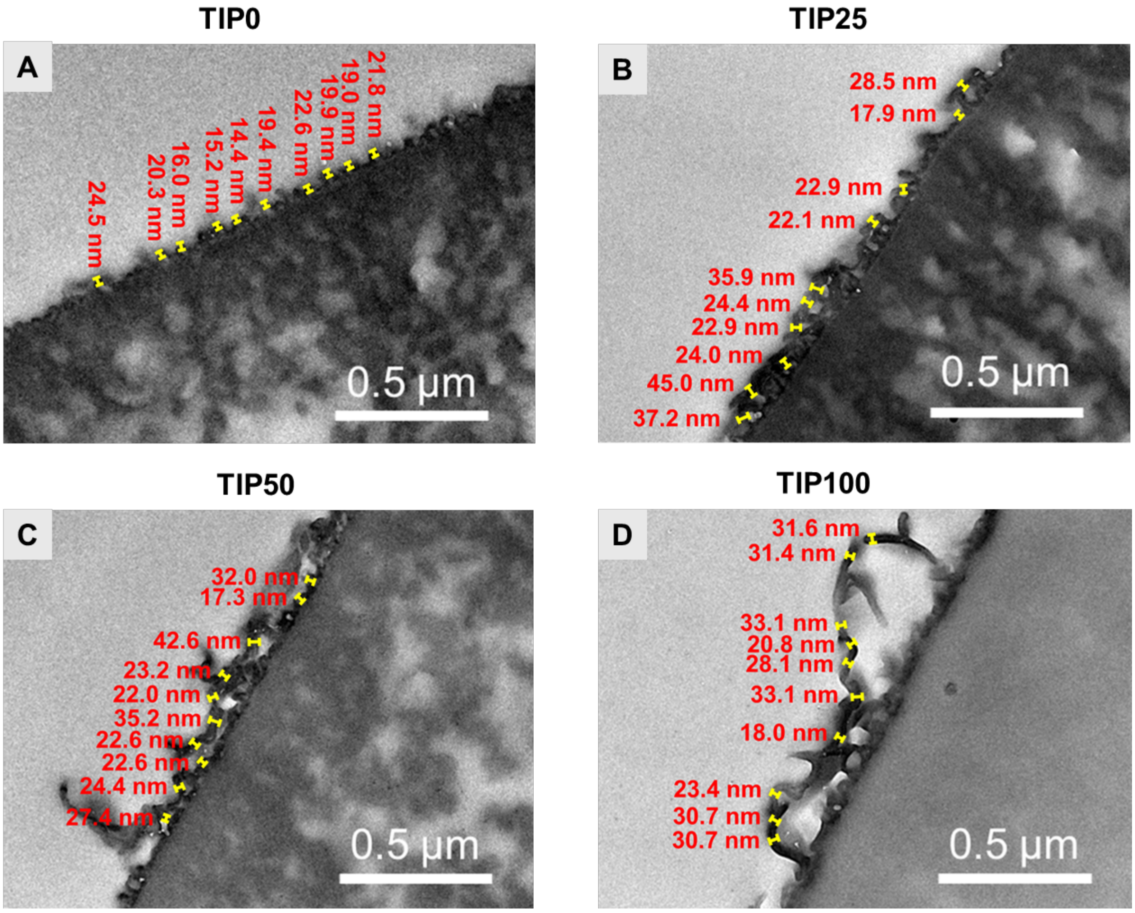

Supplementary Fig. 18| Intrinsic thickness measurement based on the TEM cross-section images of various TIP membranes: (**A**) TIP0, (**B**) TIP25, (**C**) TIP50, and (**D**) TIP100. Ten different regions were measured for each membrane sample using the software of *Image-Pro Plus* to determine the intrinsic thickness.

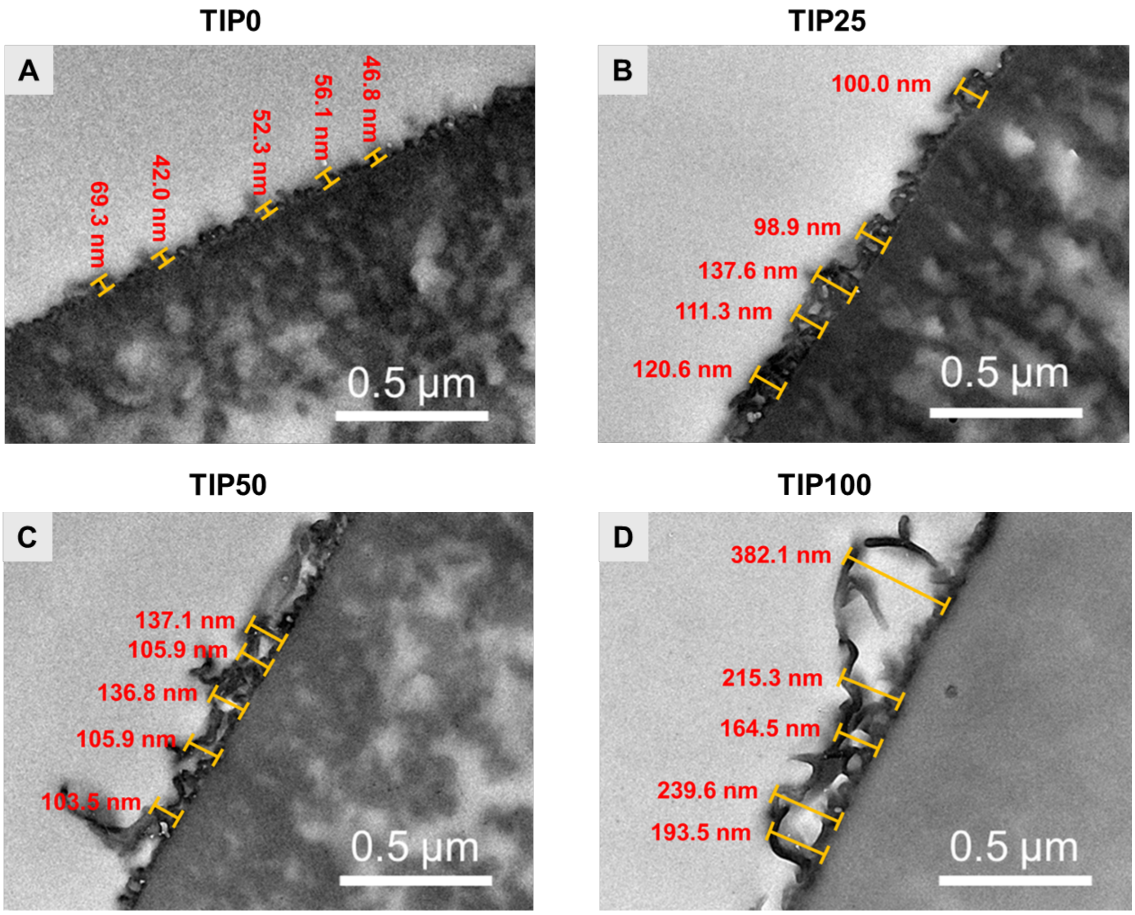

Supplementary Fig. 19| Apparent thickness measurement based on the TEM cross-section images of various TIP membranes: (**A**) TIP0, (**B**) TIP25, (**C**) TIP50, and (**D**) TIP100. Five different regions were measured for each membrane sample using the software of *Image-Pro Plus* to determine the apparent thickness.

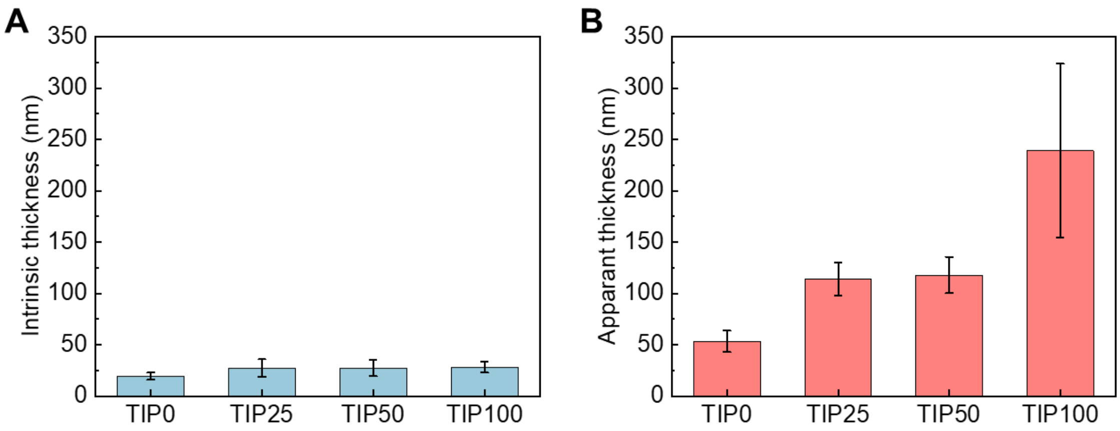

Supplementary Fig. 20| (**A**) Intrinsic thickness and (**B**) apparent thickness of various TIP membranes. The error bars represent the standard deviation of the results obtained from at least five independent measurements.

We further evaluated the intrinsic thickness (Supplementary Fig. 18) and apparent thickness (Supplementary Fig. 19) of various TIP membranes based on their TEM cross-sectional images using the software of *Image-Pro Plus*. There is no significant change in the intrinsic thickness of TIP membranes fabricated at different temperatures (Supplementary Fig. 20A). In contrast, membrane apparent thickness increased over 4 times from 53.3 nm for TIP0 to 239.0 nm for TIP100 when the reaction temperature raised from 0 °C to 100 °C (Supplementary Fig. 20B). This enhanced apparent thickness is attributed to the enlarged nanovoids within the polyamide due to the intensified interfacial degassing at higher IP temperature. As a result, the TIP membrane (e.g., TIP100) with a maintained intrinsic thickness and a higher apparent thickness possessed more effective filtration area and optimized water transport pathway, leading to enhanced water permeance (**Fig. 1A**).

Supplementary Note 16. Average roughness of TIP membranes

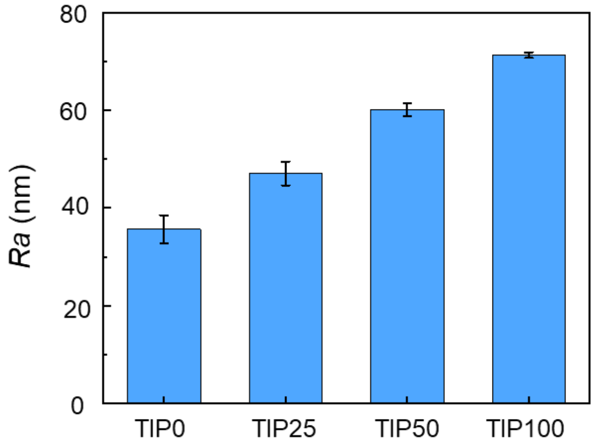

Supplementary Fig. 21| Average roughness (R_a) of various TIP membranes as determined by an atomic force microscope (AFM). The error bars represent the standard deviation of the results obtained from at least three independent measurements of different membranes.

As shown in Supplementary Fig. 21, the average roughness R_a increased from 35.7 nm for TIP0 membrane to 71.3 nm for TIP100 membrane, suggesting the significant impact of temperature on membrane surface roughness. This result is consistent with the enlarged "leaf-like" surface features (**Fig. 3A**) and extensive nanovoid structure (**Fig. 3C**) of TIP100 membrane.

Supplementary Note 17. UV-based humic acid (HA) determination

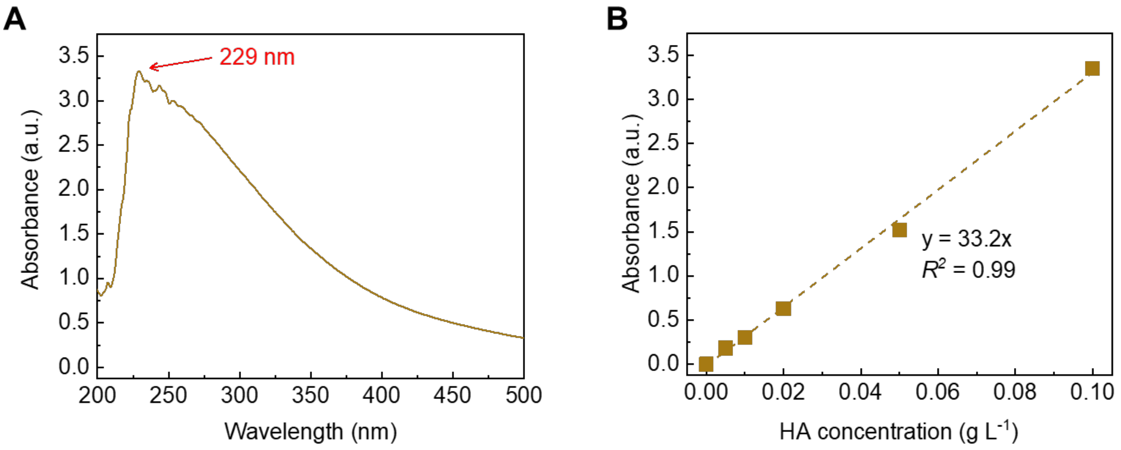

Supplementary Fig. 22| (**A**) The UV absorption spectrum of 0.1 g L^{-1} HA with a wavelength ranging from 200 to 500 nm. (**B**) Standard curve between pre-determined HA concentration (0, 0.005, 0.01, 0.02, 0.05, and 0.1 g L^{-1}) and the corresponding UV absorbance at 229 nm.

We used a UV spectrometer to determine the HA amount deposited on the membrane surface during fouling experiments. Briefly, HA was extracted by thoroughly washing the fouled membrane with an alkaline solution. The eluted solution was then analyzed by a UV spectrometer (UH5300, Hitachi) at a characteristic wavelength of 229 nm for HA (Supplementary Fig. 22A). The HA concentration was calculated based on the standard curve with a strong linear correlation (R^2 = 0.99, Supplementary Fig. 22B) between HA concentration and its corresponding UV absorbance.

Supplementary Note 18. Flux distribution of TIP membranes

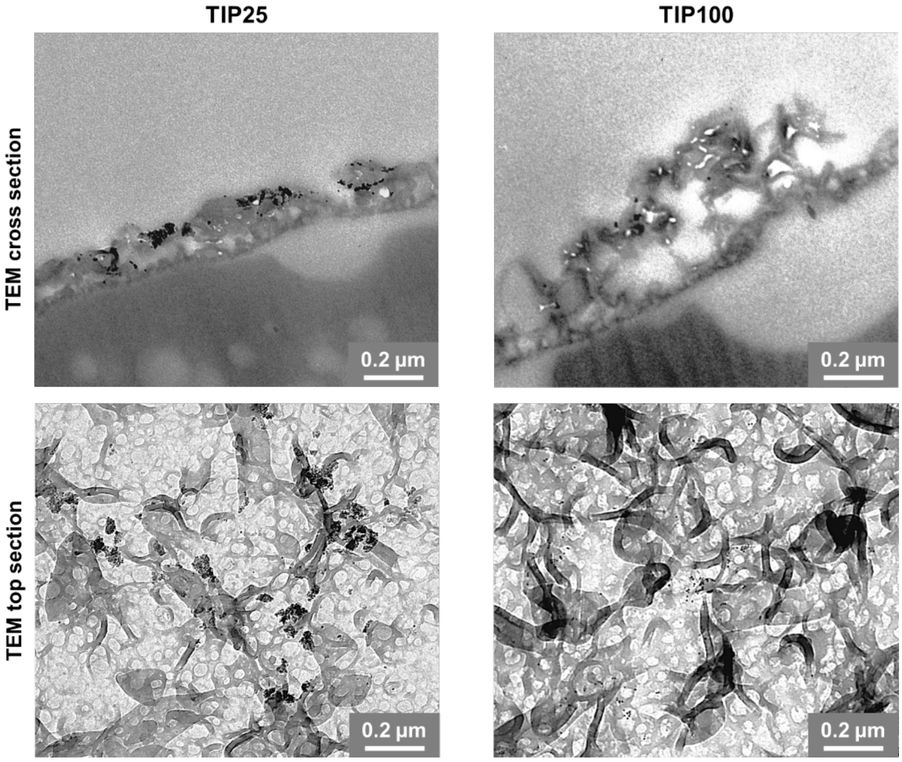

Supplementary Fig. 23| TEM cross-sectional and top-view images of TIP25 and TIP100 membranes after the filtration experiments using 5-nm gold nanoparticles as tracers. The filtration experiments were conducted using a dead-end filtration setup with a feed solution containing 1.0×10^{12} gold nanoparticles mL^{-1} at a flux of 6 L m^{-2} h^{-1} for 2 h under the room temperature of ~25 °C.

We performed additional filtration tests using gold nanoparticles (AuNPs) with a diameter of 5 nm as tracers to evaluate water transport behavior across the polyamide layer of TIP membranes. In principle, the nanosized AuNPs were likely to follow the water transport pathways and then deposit at the area with a higher localized water flux.[13-15] As shown in Supplementary Fig. 23, significant AuNPs accumulation was observed on the TIP25 membrane surface, implying the uneven flux distribution with high localized water flux. In contrast, TIP100 membrane surface showed much less deposition of AuNPs. This result could be attributed to an increased effective filtration area of TIP100 that would reduce the localized flux thereby decreasing the hydrodynamic drag force acting on the AuNPs.[16-18]

Supplementary Note 19. Molecular dynamics (MD) simulation of monomer diffusion

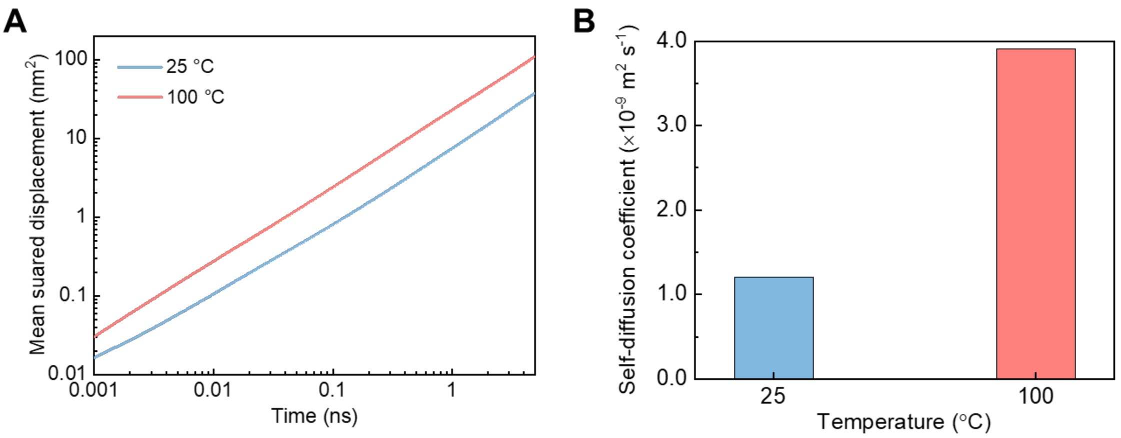

Supplementary Fig. 24| (**A**) Mean squared displacement profile versus diffusion time plotted on a log-log scale, and (**B**) self-diffusion coefficient of MPD molecules in water at 25 °C and 100 °C, respectively.

We have conducted an MD simulation to analyze MPD diffusion behavior at 25 °C and 100 °C. The aqueous and organic phase were placed into a box firstly. After reaching the equilibrated status, MPD molecules were introduced into the aqueous phase or the organic phase. The simulation duration was 20 ns and the last 5 ns simulation trajectories were adopted for further analysis. As shown in Supplementary Fig. 24, the log-log plot of mean squared displacement (MSD) versus diffusion time reveals a linear relationship (MSD $\propto$ t), confirming that the system has reached the diffusive regime. Increased temperature resulted in faster monomer diffusion that the self-diffusion coefficient improved from 1.2×10^{-9} m^2 s^{-1} to 3.9×10^{-9} m^2 s^{-1} when the temperature of aqueous phase raised from 25 °C to 100 °C.

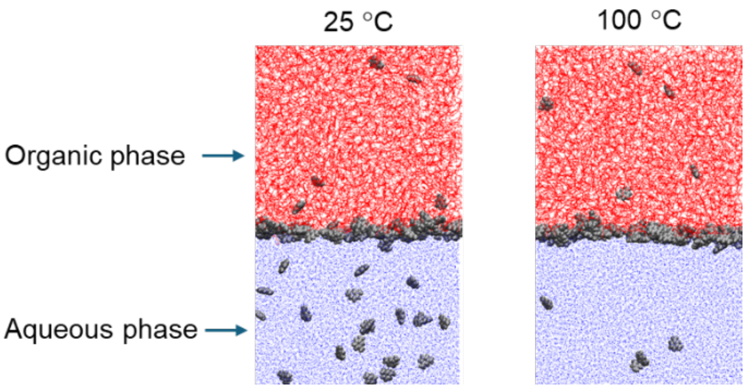

Supplementary Fig. 25| The MD simulation result of MPD (black molecules) diffusion under an equilibrium state with an organic phase temperature of 25 °C and 100 °C, respectively.

A high organic phase temperature facilitated MPD diffusion from the aqueous phase toward the aqueous/organic interface (Supplementary Fig. 25). Consequently, more MPD molecules were accumulated at the interface at 100 °C (**Fig. 5B**) thereby facilitating their diffusion into the organic phase, leading to the improved reaction with TMC.

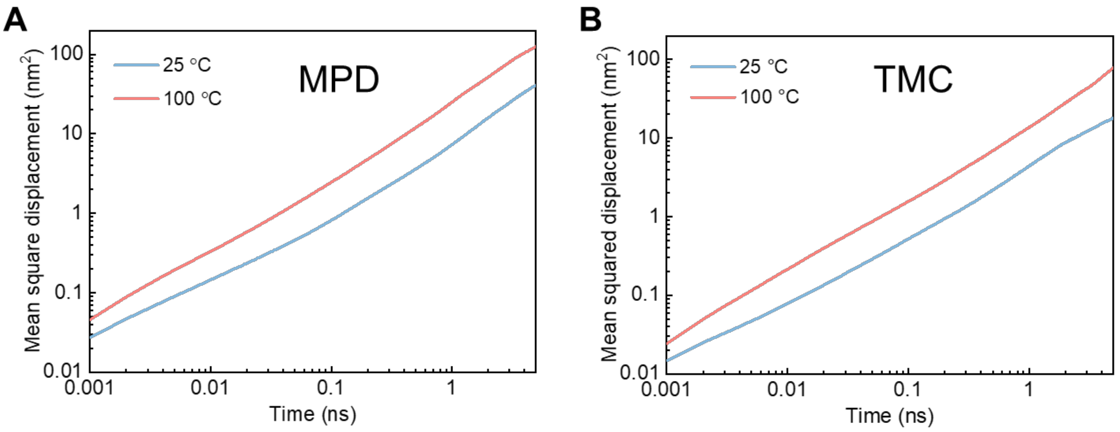

Supplementary Fig. 26| Mean squared displacement profile versus diffusion time plotted on a log-log scale for (**A**) MPD and (**B**) TMC monomers in the organic phase of Isopar G at 25 °C and 100 °C, respectively.

As shown in Supplementary Fig. 26, mean squared displacement curves for both MPD and TMC in the 100 °C Isopar G was remarkably higher than that in 25 °C. This result demonstrated

faster monomer diffusion in the organic solution with higher temperature, which would be beneficial for the IP reaction.

Supplementary Note 20. Density functional theory (DFT) calculation of IP reaction

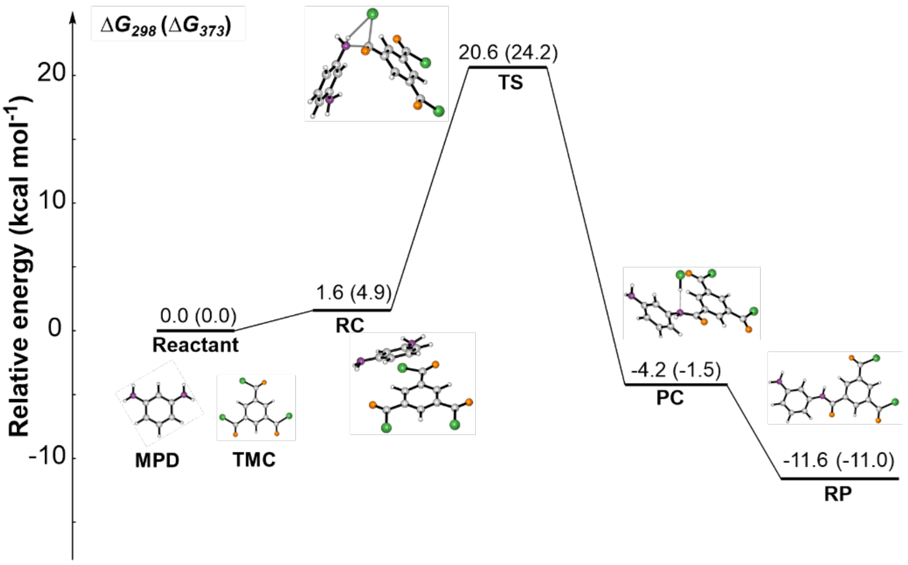

Supplementary Fig. 27| Free energy variation for the IP reaction between MPD and TMC at 25 °C (the value outside the parentheses) and 100 °C (the value inside the parentheses). The reaction was divided into five states: reactant, reaction complex (RC), transition state (TS), product complex (PC), and reaction products (RP). The energy barrier is the relative energy difference between the TS and reactant.

We performed a DFT calculation to reveal the effects of temperature on the IP reaction between MPD and TMC at a molecular level. The energy barrier of IP reaction between MPD and TMC was comparable at 25 °C and 100 °C (Supplementary Fig. 27). However, the reaction rate constant at 100 °C was much higher than that at 25 °C (0.99 s^{-1} vs. 0.05 s^{-1}, **Fig. 5D**). The higher reaction rate could accelerate the IP reaction to form a more crosslinked polyamide layer with extensive nanostructures.

Supplementary Note 21. Chemical information about micropollutants

Supplementary Table 3| The chemical formula, structure, and molecular weight of various organic micropollutants used in this study.

**Micropollutant**	**Formula**	**Structure**	**Molecular weight (g mol^{-1})**
Methylparaben	$C_8H_8O_3$	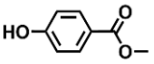	152.1
Ethylparaben	$C_9H_{10}O_3$	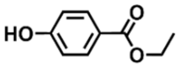	166.2
Propylparaben	$C_{10}H_{12}O_3$	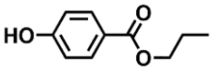	180.2
Benzylparaben	$C_{14}H_{12}O_3$	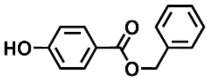	228.2
Sulfadiazine	$C_{10}H_{10}N_4O_2S$	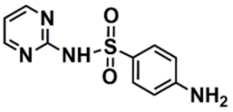	250.3
Sulfamethoxazole	$C_{10}H_{11}N_3O_3S$	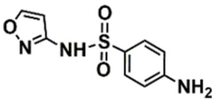	253.3
Sulfamethazine	$C_{12}H_{14}N_4O_2S$	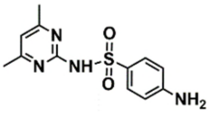	278.3
Norfloxacin	$C_{16}H_{18}FN_3O_3$	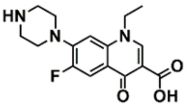	319.3
Ofloxacin	$C_{18}H_{20}FN_3O_4$	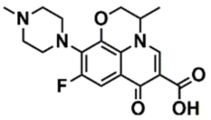	361.3

Supplementary Note 22. Rejection of micropollutants by polyamide membranes

Supplementary Table 4| Rejection behaviour of boron by polyamide membranes at a neutral pH of 6-8.

Membrane	*A* (L m^{-2} h^{-1} bar^{-1})	*R* (%)	*B* (L m^{-2} h^{-1})	*A/B* (bar^{-1})	Reference
RO-0.5	1.17	60.9	13.14	0.09	[19]
RO-1	1.15	73.5	7.28	0.16	[19]
RO-5	1.19	77.1	6.19	0.19	[19]
RO-10	1.19	80.2	5.14	0.23	[19]
RO-60	1.18	83.5	4.07	0.29	[19]
KRO-1	0.75	84	2.21	0.34	[20]
RO4	0.62	69	15.38	0.04	[20]
UTC-80LB	0.83	72	5.00	0.17	[21]
BW30	0.35	75	5.83	0.60	[22]
AG	N.A.	62	N.A.	N.A.	[22]
ESPA2	2.57	82	13.54	0.19	[23]
ESPAB	2.96	69	31.92	0.09	[23]
BW30LE	2.14	71	20.98	0.10	[23]
TFN	1.7	71	6.11	0.28	[24]
TFC	0.9	87	2.25	0.40	[24]
RO1	6.56	44.4	74.59	0.09	[25]
RO2	1.88	65.4	10.00	0.19	[25]
FO1	2.33	39.2	804.02	0.01	[25]
TFC-hex	0.82	67.6	5.88	0.14	[26]
TFC-hep	0.82	63.1	7.17	0.11	[26]
TFC-cyclo	0.81	69.3	5.35	0.15	[26]
TFC-isopar	0.60	74.3	3.11	0.19	[26]
TFC-hex-r	0.98	66.1	7.52	0.13	[26]
TFC-hep-r	0.96	69.2	6.38	0.15	[26]
TFC-cyclo-r	0.98	64.7	8.00	0.12	[26]
TFC-isopar-r	0.97	69.8	6.27	0.15	[26]
TFC-pH4.0	0.52	49	8.33	0.06	[27]
TFC-pH5.0	1.35	69	9.43	0.14	[27]
TFC-pH6.3	1.68	73	9.62	0.17	[27]
TFC-pH9.3	2.00	77	9.26	0.22	[27]
TFC-pH10.3	2.19	79	9.04	0.24	[27]
TFC-pH12.5	2.39	71	15.11	0.16	[27]
TFC-0.5	6.20	68	2.92	0.14	[28]
TFC-2.0	29.45	71	11.45	0.17	[28]
TFC-8.0	17.05	82	3.74	0.29	[28]
SW30	1.00	87	3.81	0.26	[29]

Continued on next page

(**Supplementary Table 4** *Continued*)

**Membrane**	***A*** **(L m^{-2} h^{-1} bar^{-1})**	***R*** **(%)**	***B*** **(L m^{-2} h^{-1})**	***A/B*** **(bar^{-1})**	**Reference**
UTC-80	0.58	87.8	3.88	0.15	[30]
TW30	2.90	63	36.23	0.08	[31]
PIB	1.61	88.2	3.34	0.48	[32]
TFC-TOB_2/APD1	2.56	87.6	5.80	0.44	[33]
BW30FR	2.90	67	22.14	0.13	[34]
BW30XFR	3.10	72	18.69	0.17	[34]
TMG800M	N.A.	80	N.A.	N.A.	[35]
TFC-TFPD	1.25	86.6	9.67	0.13	[36]
TFC-TFBC	1.27	87	9.49	0.13	[36]
TFC-TFEA	0.80	87.5	5.71	0.14	[36]
TFN-0.1	2.70	82	9.19	0.29	[37]
SRN	1.46	62	13.42	0.11	[38]
TR-BE	1.25	49	26.02	0.05	[39]
SWC5	N.A.	82	N.A.	N.A.	[40]
PA@NS	1.1	77	18.14	0.06	[41]
PA@TD	0.9	84	9.46	0.10	[41]
SW30HR	0.6	89	4.09	0.15	[42]

Supplementary Table 5| Rejection behaviour of As (III) by polyamide membranes at a neutral pH of 6-8.

Membrane	*A* (L m^{-2} h^{-1} bar^{-1})	*R* (%)	*B* (L m^{-2} h^{-1})	*A/B* (bar^{-1})	Reference
BW30LE	1.0	80.3	9.81	0.10	[43]
TFC-pH10.3	2.4	93.4	2.37	1.01	[27]
TFC-pH12.5	2.7	88.5	4.85	0.56	[27]
TFC-0.5	0.8	85.7	2.05	0.39	[28]
TFC-2	1.8	88.1	3.75	0.48	[28]
TFC-8	1.1	89.6	1.96	0.56	[28]
TR-BE	1.17	91.0	2.31	0.51	[39]
ES10	0.27	76.3	1.27	0.21	[44]
NTR-729HF	0.23	23.4	11.1	0.02	[44]
TW	2.78	89.1	3.27	0.85	[45]
SW	1.70	96.3	0.99	1.71	[45]
BW30	0.2	60.8	0.28	0.18	[46]
SWHR	0.2	77.4	2.06	0.17	[46]
TFC	N.A.	61.5	N.A.	N.A.	[47]
TFC-ULPT	N.A.	75.9	N.A.	N.A.	[47]
AG	N.A.	70.3	N.A.	N.A.	[47]
LSA-CPA2	N.A.	87.2	N.A.	N.A.	[47]
TFC-S	2.7	64.2	13.55	0.20	[48]
ESPA4	2.57	77.6	6.67	0.38	[48]
RO4	1.5	94.6	1.33	1.13	[49]
Toray UTC70	0.4	74.3	0.76	0.53	[50]
HR	N.A.	90.5	N.A.	N.A.	[51]
Desal AK	7.57	64.5	28.73	0.26	[52]
AD	0.53	94.2	0.33	1.62	[53]
BE	2	90	2.22	0.90	[53]
UTC 80 B	0.9	95.2	0.45	1.98	[53]
TFM-100	N.A.	50	N.A.	N.A.	[54]
osm-ESPA	2.49	60	53.13	0.05	[55]
Base-PA	2.91	91.8	3.02	0.96	[56]
Toray SWRO	1.33	92.4	3.8	0.35	[56]
SNS	1.69	95.8	1.06	1.59	[56]
SW30HR	0.87	96.0	0.2	4.35	[23]
H_PAMAM G2	2.9	83.1	5.90	0.49	[57]
H_TFC	2.3	89.1	2.81	0.82	[57]
TW30	0.19	79.3	0.73	0.26	[58]

Supplementary Table 6| Rejection of endocrine disrupting compounds (EDCs) by polyamide membranes.

Membrane	Compound	Rejection (%)	Reference
BW30	17β-estradiol	96	[59]
BW30	17α-ethynylestradiol	95	[59]
BW30	Bisphenol A	98	[59]
BW30	Progesterone	94	[59]
ESPA2	Bisphenol A	95	[59]
ESPA1	4-nonylphenol	86	[60]
ESPA1	Bisphenol A	96	[60]
LFC1	4-nonylphenol	85	[60]
GE Osmonics AK	Estrone	90	[61]
KOCH TFC-S	Estrone	95	[62]
LE	Octylphenol	78.1	[63]
LE	4-nonylphenol	94.0	[63]
Trisep X20	Bisphenol A	96.1	[64]
Trisep X20	Diethyltoluamide	96.1	[64]
Trisep X20	Equilin	97.3	[64]
Trisep X20	17α-estradiol	96.6	[64]
Trisep X20	17β-estradiol	95.6	[64]
Trisep X20	Estradiol	97.1	[64]
X20	Alachlor	96.7	[64]
X20	Atraton	97.3	[64]
X20	Bisphenol A	96.1	[64]
X20	Diethyltoluamide	96.1	[64]
X20	17α-estradiol	96.8	[64]
X20	17β-estradiol	95.6	[64]
X20	Estriol	95.2	[64]
X20	Estrone	96.9	[64]
XLE	Carbaryl	79	[65]
XLE	4-phenylphenol	61	[65]
XLE	Methylparaben	53	[65]
XLE	Ethylparaben	76	[65]
XLE	Propylparaben	77	[66]
XLE	Benzylparaben	82	[66]
XLE	Bisphenol A	83	[66]
XLE	17β-estradiol	83	[66]
TW30	Bisphenol A	87	[67]
CE BWRO	Bisphenol A	84	[67]
PA/TNT TFC	Bisphenol A	89.1	[67]
UTC-60	Bisphenol A	36	[68]
UTC-60	17β-estradiol	57	[68]
UTC-60	4-nonylphenol	82	[68]

Supplementary Table S7| Rejection of antibiotics by polyamide membranes.

Membrane	Compound	Rejection (%)	Reference
BW30	Acetaminophen	72	[59]
ESPA2	Phenacetine	82	[59]
ESPA2	Caffeine	90	[59]
ESPA2	Carbamazepine	95	[59]
KOCH TFC-S	Gemfibrozil	97.4	[63]
KOCH TFC-S	Aalicylic acid	97.7	[63]
KOCH TFC-S	Tris(1-chloro-2-propyl) phosphate	22.5	[63]
LE	Vardenafil	59.1	[63]
LE	Tadalafil	98.1	[63]
LE	Phantolide	88.9	[63]
LE	Tonalide	98.0	[63]
Trisep X20	Acetaminophen	92.2	[64]
Trisep X20	Caffeine	94.7	[64]
Trisep X20	Carbadox	95.3	[64]
Trisep X20	Carbamazepine	91.5	[64]
Trisep X20	Gemfibrozil	96.9	[64]
Trisep X20	Sulfachloropyridazine	96.3	[64]
Trisep X20	Sulfamerazine	94.4	[64]
Trisep X20	Sulfamethoxazole	95.6	[64]
X20	Caffeine	97.0	[64]
X20	Carbadox	96.2	[64]
X20	Carbamazepine	97.0	[64]
X20	Gemfibrozil	98.5	[64]
X20	Metolachlor	97.4	[64]
X20	Sulfamerazine	97.2	[64]
X20	Sulfamethoxazole	98.8	[64]
XLE	Phenacetine	74	[65]
XLE	Caffeine	70	[65]
XLE	Primidone	87	[65]
XLE	Isopropylantipyrine	78	[65]
XLE	Carbamazepine	91	[65]
XLE	Sulfamethoxazole	70	[65]
TFC	Caffeine	95	[67]
SW30	Carbamazepine	98	[69]
BW30	Carbamazepine	98	[69]
BW30	Acetaminophen	72	[70]
BW30	Caffeine	98	[70]
BW30	Cotinine	98	[70]
BW30	Ibuprofen	98	[70]
BW30	Sulfamethoxazole	92	[70]

Continued on next page

(**Supplementary Table 7** *Continued*)

**Membrane**	**Compound**	**Rejection (%)**	**Reference**
BW30	Triclosan	98	[70]
BW30	Trimethoprim	87	[70]
ESPA1	Atenolol	97.5	[71]
ESPA1	Metoprolol	97.1	[71]
ESPA1	Trimethoprim	96.0	[71]
ESPA1	Propranolol	94.8	[71]
ESPA1	Carbamazepine	98.7	[71]
ESPA1	Primidone	98.6	[71]
ESPA2	Atenolol	94.4	[72]
ESPA2	Tris(2-chloroethyl) phosphate	96.3	[72]
GE Osmonics AK	Cyclophosphamide	94	[73]
LFC1	Gemfibrozil	98.8	[74]
LFC1	Hydrochlorothiazide	94.5	[74]
LFC1	Ibuprofen	96.7	[74]
LFC1	Naproxen	98.3	[74]
LFC1	Nicotine	76	[74]
LFC1	Ofloxacin	95.4	[74]
TFC-HR	Naproxen	97	[75]
TFC-HR	Diclofenac	94	[75]
TFC-HR	Ibuprofen	95	[75]
TFC-HR	Mecoprop	98	[75]
TFC-HR	Ketoprofen	97	[75]
TFC-HR	Gemfibrozil	90	[75]
TFC-HR	Primidone	91	[75]
RE8040-FL	Atenolol	86.5	[76]
RE8040-FL	Carbamazepine	98.4	[76]
RE8040-FL	Caffeine	65.4	[76]
RE8040-FL	Dilatin	90.9	[76]
SR	Atenolol	98	[77]
SR	Sulfamethoxazole	97	[77]
SR	Caffeine	97	[77]
SR	Trimethoprim	98	[77]
SR	Carbamazepine	98	[77]
SR	Amitriptyline	89	[77]
SR	Primidone	62	[77]
SR	Verapamil	86	[77]
SR	Diclofenac	97	[77]
SR	Naproxen	98	[77]
XLE	Trimethoprim	98.6	[78]

Supplementary References

1. Tang, C.Y., Kwon, Y.-N. & Leckie, J.O. Effect of membrane chemistry and coating layer on physiochemical properties of thin film composite polyamide RO and NF membranes: I. FTIR and XPS characterization of polyamide and coating layer chemistry. *Desalination* **242**, 149-167 (2009).
2. Youm, K.H. & Kim, W.S. Prediction of intrinsic pore properties of ultrafiltration membrane by solute rejection curves: effects of operating conditions on pore properties. *J. Chem. Eng. Japan* **24**, 1-7 (1991).
3. Van der Bruggen, B. & Vandecasteele, C. Modelling of the retention of uncharged molecules with nanofiltration. *Water Res.* **36**, 1360-1368 (2002).
4. Yang, Z. et al. Mechanistic insights into the role of polydopamine interlayer toward improved separation performance of polyamide nanofiltration membranes. *Environ. Sci. Technol.* **54**, 11611-11621 (2020).
5. Deen, W.M. Hindered transport of large molecules in liquid-filled pores. *AIChE Journal* **33**, 1409-1425 (1987).
6. Zhao, C. et al. Polyamide membranes with nanoscale ordered structures for fast permeation and highly selective ion-ion separation. *Nat. Commun.* **14**, 1112 (2023).
7. Peng, L.E. et al. Does interfacial vaporization of organic solvent affect the structure and separation properties of polyamide RO membranes? *J. Membr. Sci.* **625**, 119173 (2021).
8. Song, X., Gan, B., Yang, Z., Tang, C.Y. & Gao, C. Confined nanobubbles shape the surface roughness structures of thin film composite polyamide desalination membranes. *J. Membr. Sci.* **582**, 342-349 (2019).
9. Gan, Q. et al. Nanofoamed polyamide membranes: mechanisms, developments, and environmental implications. *Environ. Sci. Technol.* **58**, 20812-20829 (2024).
10. Song, X. et al. Intrinsic nanoscale structure of thin film composite polyamide membranes: connectivity, defects, and structure–property correlation. *Environ. Sci. Technol.* **54**, 3559-3569 (2020).
11. Davenport, D.M. et al. Thin film composite membrane compaction in high-pressure reverse osmosis. *J. Membr. Sci.* **610**, 118268 (2020).
12. Davenport, D.M., Wang, L., Shalusky, E. & Elimelech, M. Design principles and challenges of bench-scale high-pressure reverse osmosis up to 150 bar. *Desalination* **517**, 115237 (2021).
13. Pacheco, F., Sougrat, R., Reinhard, M., Leckie, J.O. & Pinnau, I. 3D visualization of the internal nanostructure of polyamide thin films in RO membranes. *J. Membr. Sci.* **501**, 33-44 (2016).
14. Li, Y. et al. Probing flow activity in polyamide layer of reverse osmosis membrane with nanoparticle tracers. *J. Membr. Sci.* **534**, 9-17 (2017).
15. Pacheco, F.A., Pinnau, I., Reinhard, M. & Leckie, J.O. Characterization of isolated polyamide thin films of RO and NF membranes using novel TEM techniques. *J. Membr. Sci.* **358**, 51-59 (2010).
16. Tang, C.Y., Kwon, Y.-N. & Leckie, J.O. Fouling of reverse osmosis and nanofiltration membranes by humic acid—Effects of solution composition and hydrodynamic conditions. *J. Membr. Sci.* **290**, 86-94 (2007).
17. Ramon, G.Z., Wong, M.C.Y. & Hoek, E.M.V. Transport through composite membrane, part 1: is there an optimal support membrane? *J. Membr. Sci.* **415-416**, 298-305 (2012).
18. Ramon, G.Z. & Hoek, E.M.V. Transport through composite membranes, part 2: impacts of roughness on permeability and fouling. *J. Membr. Sci.* **425-426**, 141-148 (2013).
19. Zhou, S. et al. Unveiling the growth of polyamide nanofilms at water/organic free interfaces: toward enhanced water/salt selectivity. *Environ. Sci. Technol.* **56**, 10279-10288 (2022).
20. Ali, Z. et al. Defect-free highly selective polyamide thin-film composite membranes for desalination and boron removal. *J. Membr. Sci.* **578**, 85-94 (2019).

21. Taniguchi, M., Kurihara, M. & Kimura, S. Boron reduction performance of reverse osmosis seawater desalination process. *J. Membr. Sci.* **183**, 259-267 (2001).
22. Cengeloglu, Y., Arslan, G., Tor, A., Kocak, I. & Dursun, N. Removal of boron from water by using reverse osmosis. *Sep. Purif. Technol.* **64**, 141-146 (2008).
23. Teychene, B., Collet, G., Gallard, H. & Croue, J.-P. A comparative study of boron and arsenic (III) rejection from brackish water by reverse osmosis membranes. *Desalination* **310**, 109-114 (2013).
24. Hofs, B. et al. Characterization and performance of a commercial thin film nanocomposite seawater reverse osmosis membrane and comparison with a thin film composite. *J. Membr. Sci.* **446**, 68-78 (2013).
25. Kim, S.-J. et al. Performance evaluation of polyamide TFC membranes: effects of free volume properties on boron transport. *Desalination* **432**, 104-114 (2018).
26. Chong, C.Y. et al. Studies on the properties of RO membranes for salt and boron removal: influence of thermal treatment methods and rinsing treatments. *Desalination* **428**, 218-226 (2018).
27. Peng, L.E. et al. Tailoring polyamide rejection layer with aqueous carbonate chemistry for enhanced membrane separation: mechanistic insights, chemistry-structure-property relationship, and environmental implications. *Environ. Sci. Technol.* **53**, 9764-9770 (2019).
28. Peng, L.E. et al. Deciphering the role of amine concentration on polyamide formation toward enhanced RO performance. *ACS EST Engg.* **2**, 903-912 (2022).
29. Tu, K.L., Nghiem, L.D. & Chivas, A.R. Coupling effects of feed solution pH and ionic strength on the rejection of boron by NF/RO membranes. *Chem. Eng. J.* **168**, 700-706 (2011).
30. Koseoglu, H., Kabay, N., Yüksel, M. & Kitis, M. The removal of boron from model solutions and seawater using reverse osmosis membranes. *Desalination* **223**, 126-133 (2008).
31. Dydo, P., Turek, M., Ciba, J., Trojanowska, J. & Kluczka, J. Boron removal from landfill leachate by means of nanofiltration and reverse osmosis. *Desalination* **185**, 131-137 (2005).
32. Wang, S., Zhou, Y. & Gao, C. Novel high boron removal polyamide reverse osmosis membranes. *J. Membr. Sci.* **554**, 244-252 (2018).
33. Li, J., Wang, D., Wang, Y., Gao, B. & Wang, Z. Surface modification of reverse osmosis membranes for enhanced boron removal and fouling resistance. *ACS ES&T Water* **1**, 2284-2292 (2021).
34. Chen, D., Zhao, X. & Li, F. Influence of boron on rejection of trace nuclides by reverse osmosis. *Desalination* **370**, 72-78 (2015).
35. Breitner, L.N., Howe, K.J. & Minakata, D. Boron can be used to predict trace organic rejection through reverse osmosis membranes for potable reuse. *Environ. Sci. Technol.* **52**, 13871-13878 (2018).
36. Li, C., Zhao, Y., Lai, G.S. & Wang, R. Fabrication of fluorinated polyamide seawater reverse osmosis membrane with enhanced boron removal. *J. Membr. Sci.* **662**, 121009 (2022).
37. Liu, L. et al. Thin film nanocomposite reverse osmosis membrane incorporated with UiO-66 nanoparticles for enhanced boron removal. *J. Membr. Sci.* **580**, 101-109 (2019).
38. Di Vincenzo, M., Barboiu, M., Tiraferri, A. & Legrand, Y.M. Polyol-functionalized thin-film composite membranes with improved transport properties and boron removal in reverse osmosis. *J. Membr. Sci.* **540**, 71-77 (2017).
39. Jarma, Y.A. et al. Assessment of different nanofiltration and reverse osmosis membranes for simultaneous removal of arsenic and boron from spent geothermal water. *J. Hazard. Mater.* **405**, 124129 (2021).
40. Tu, K.L., Chivas, A.R. & Nghiem, L.D. Effects of chemical preservation on flux and solute rejection by reverse osmosis membranes. *J. Membr. Sci.* **472**, 202-209 (2014).
41. Wang, S. et al. Surface engineering design of polyamide membranes for enhanced boron removal in seawater desalination. *J. Membr. Sci.* **651**, 120425 (2022).

42. Koseoglu, H. et al. Boron removal from seawater using high rejection SWRO membranes — impact of pH, feed concentration, pressure, and cross-flow velocity. *Desalination* **227**, 253-263 (2008).
43. Regis, A.O. et al. Pressure-driven membrane processes for boron and arsenic removal: pH and synergistic effects. *Desalination* **522**, 115441 (2022).
44. Kang, M., Kawasaki, M., Tamada, S., Kamei, T. & Magara, Y. Effect of pH on the removal of arsenic and antimony using reverse osmosis membranes. *Desalination* **131**, 293-298 (2000).
45. Geucke, T., Deowan, S.A., Hoinkis, J. & Pätzold, C. Performance of a small-scale RO desalinator for arsenic removal. *Desalination* **239**, 198-206 (2009).
46. Akin, I., Arslan, G., Tor, A., Cengeloglu, Y. & Ersoz, M. Removal of arsenate [As(V)] and arsenite [As(III)] from water by SWHR and BW-30 reverse osmosis. *Desalination* **281**, 88-92 (2011).
47. Brandhuber, P. & Amy, G. Alternative methods for membrane filtration of arsenic from drinking water. *Desalination* **117**, 1-10 (1998).
48. Richards, L.A., Richards, B.S. & Schäfer, A.I. Renewable energy powered membrane technology: salt and inorganic contaminant removal by nanofiltration/reverse osmosis. *J. Membr. Sci.* **369**, 188-195 (2011).
49. Aljubran, M.A. et al. Highly efficient size-sieving-based removal of arsenic(III) via defect-free interfacially-polymerized polyamide thin-film composite membranes. *J. Membr. Sci.* **652**, 120477 (2022).
50. Ahmed, S., Rasul, M.G., Hasib, M.A. & Watanabe, Y. Performance of nanofiltration membrane in a vibrating module (VSEP-NF) for arsenic removal. *Desalination* **252**, 127-134 (2010).
51. Oh, J.I. et al. Application of low-pressure nanofiltration coupled with a bicycle pump for the treatment of arsenic-contaminated groundwater. *Desalination* **132**, 307-314 (2000).
52. Chang, F.-f., Liu, W.-j. & Wang, X.-m. Comparison of polyamide nanofiltration and low-pressure reverse osmosis membranes on As(III) rejection under various operational conditions. *Desalination* **334**, 10-16 (2014).
53. Abejón, A., Garea, A. & Irabien, A. Arsenic removal from drinking water by reverse osmosis: minimization of costs and energy consumption. *Sep. Purif. Technol.* **144**, 46-53 (2015).
54. Chan, B.K.C. & Dudeney, A.W.L. Reverse osmosis removal of arsenic residues from bioleaching of refractory gold concentrates. *Miner. Eng.* **21**, 272-278 (2008).
55. Dao, D.T., Duong, H.C., Laborie, S. & Cabassud, C. Integrated reverse osmosis/vacuum membrane distillation for enhanced arsenic removal and water recovery of brackish water desalination. *Desalin. Water Treat.* **287**, 19-28 (2023).
56. Chen, Y. & Cohen, Y. RO membrane with a surface tethered polymer brush layer for enhanced rejection of nitrate, boron, and arsenic. *J. Membr. Sci. Lett.* **3**, 100062 (2023).
57. Zhu, W.-P., Gao, J., Sun, S.-P., Zhang, S. & Chung, T.-S. Poly(amidoamine) dendrimer (PAMAM) grafted on thin film composite (TFC) nanofiltration (NF) hollow fiber membranes for heavy metal removal. *J. Membr. Sci.* **487**, 117-126 (2015).
58. Víctor-Ortega, M.D. & Ratnaweera, H.C. Double filtration as an effective system for removal of arsenate and arsenite from drinking water through reverse osmosis. *Process Saf. Environ. Prot.* **111**, 399-408 (2017).
59. Yangali-Quintanilla, V., Maeng, S.K., Fujioka, T., Kennedy, M. & Amy, G. Proposing nanofiltration as acceptable barrier for organic contaminants in water reuse. *J. Membr. Sci.* **362**, 334-345 (2010).
60. Wintgens, T., Gallenkemper, M. & Melin, T. Removal of endocrine disrupting compounds with membrane processes in wastewater treatment and reuse. *Water Sci. Technol.* **50**, 1-8 (2004).
61. Jin, X., Hu, J. & Ong, S.L. Removal of natural hormone estrone from secondary effluents using nanofiltration and reverse osmosis. *Water Res.* **44**, 638-648 (2010).

62. Schäfer, A.I., Nghiem, L.D. & Waite, T.D. Removal of the natural hormone estrone from aqueous solutions using nanofiltration and reverse osmosis. *Environ. Sci. Technol.* **37**, 182-188 (2003).
63. Gomez, V., Majamaa, K., Pocurull, E. & Borrull, F. Determination and occurrence of organic micropollutants in reverse osmosis treatment for advanced water reuse. *Water Sci. Technol.* **66**, 61-71 (2012).
64. Comerton, A.M., Andrews, R.C., Bagley, D.M. & Hao, C. The rejection of endocrine disrupting and pharmaceutically active compounds by NF and RO membranes as a function of compound and water matrix properties. *J. Membr. Sci.* **313**, 323-335 (2008).
65. Kimura, K., Toshima, S., Amy, G. & Watanabe, Y. Rejection of neutral endocrine disrupting compounds (EDCs) and pharmaceutical active compounds (PhACs) by RO membranes. *J. Membr. Sci.* **245**, 71-78 (2004).
66. Guo, H. et al. Non-polyamide based nanofiltration membranes using green metal–organic coordination complexes: implications for the removal of trace organic contaminants. *Environ. Sci. Technol.* **53**, 2688-2694 (2019).
67. Ahmad, N.A. et al. Enhanced removal of endocrine-disrupting compounds from wastewater using reverse osmosis membrane with titania nanotube-constructed nanochannels. *Membranes* **12**, 958 (2022).
68. Ozaki, H., Ikejima, N. & Matsui, S. Effect of coexisting natural organic matters (NOM) on the rejection of endocrine disrupting chemicals (EDCs) by a low pressure reverse osmosis (LPRO) membrane. *J. Appl. Membrane Sci. Techno.* **1** (2017).
69. Gur-Reznik, S., Koren-Menashe, I., Heller-Grossman, L., Rufel, O. & Dosoretz, C.G. Influence of seasonal and operating conditions on the rejection of pharmaceutical active compounds by RO and NF membranes. *Desalination* **277**, 250-256 (2011).
70. Huang, H., Cho, H., Schwab, K. & Jacangelo, J.G. Effects of feedwater pretreatment on the removal of organic microconstituents by a low fouling reverse osmosis membrane. *Desalination* **281**, 446-454 (2011).
71. Liu, Y.-l., Wang, X.-m., Yang, H.-w. & Xie, Y.F. Quantifying the influence of solute-membrane interactions on adsorption and rejection of pharmaceuticals by NF/RO membranes. *J. Membr. Sci.* **551**, 37-46 (2018).
72. Bellona, C., Heil, D., Yu, C., Fu, P. & Drewes, J.E. The pros and cons of using nanofiltration in lieu of reverse osmosis for indirect potable reuse applications. *Sep. Purif. Technol.* **85**, 69-76 (2012).
73. Wang, L. et al. Cyclophosphamide removal from water by nanofiltration and reverse osmosis membrane. *Water Res.* **43**, 4115-4122 (2009).
74. Urtiaga, A.M., Pérez, G., Ibáñez, R. & Ortiz, I. Removal of pharmaceuticals from a WWTP secondary effluent by ultrafiltration/reverse osmosis followed by electrochemical oxidation of the RO concentrate. *Desalination* **331**, 26-34 (2013).
75. Xu, P. et al. Rejection of emerging organic micropollutants in nanofiltration-reverse osmosis membrane applications. *Water Environ. Res.* **77**, 40-48 (2005).
76. Chon, K., Cho, J. & Shon, H.K. A pilot-scale hybrid municipal wastewater reclamation system using combined coagulation and disk filtration, ultrafiltration, and reverse osmosis: removal of nutrients and micropollutants, and characterization of membrane foulants. *Bioresour. Technol.* **141**, 109-116 (2013).
77. Shanmuganathan, S., Vigneswaran, S., Nguyen, T.V., Loganathan, P. & Kandasamy, J. Use of nanofiltration and reverse osmosis in reclaiming micro-filtered biologically treated sewage effluent for irrigation. *Desalination* **364**, 119-125 (2015).
78. Košutić, K., Dolar, D., Ašperger, D. & Kunst, B. Removal of antibiotics from a model wastewater by RO/NF membranes. *Sep. Purif. Technol.* **53**, 244-249 (2007).
